# Editorial: Mathematical and Statistics Anxiety: Educational, Social, Developmental and Cognitive Perspectives

**DOI:** 10.3389/fpsyg.2016.01083

**Published:** 2016-07-21

**Authors:** Kinga Morsanyi, Irene C. Mammarella, Dénes Szücs, Carlo Tomasetto, Caterina Primi, Erin A. Maloney

**Affiliations:** ^1^School of Psychology, Queen's University BelfastBelfast, UK; ^2^Department of Developmental Psychology, University of PadovaPadova, Italy; ^3^Department of Experimental Psychology, University of CambridgeCambridge, UK; ^4^Department of Psychology, University of BolognaBologna, Italy; ^5^Neurofarba–Section of Psychology, University of FlorenceFlorence, Italy; ^6^Department of Psychology, University of ChicagoChicago, IL, USA

**Keywords:** anxiety specificity, developmental change, emotional factors, gender differences, mathematical anxiety, mathematics performance, measurement

Mathematical anxiety (MA) is a feeling of apprehension and fear related to mathematics (e.g., Ashcraft, 2002). High levels of MA have serious implications for a person's life prospects, as they can lead to an avoidance of elective maths courses, which, in turn, affects people's career opportunities (e.g., Hembree, 1990). The societal importance of MA is also underlined by the fact that, according to the latest report of the Organization for Economic Co-operation and Development, on average 30.6% of adolescents get very nervous when they have to do maths problems (OECD, 2015).

Research in this area has shown an exponential growth in recent years, with the number of papers dealing with MA increasing from 60 in the year 2000 to 330 papers published in 2015 (based on Scopus statistics accessed on 20/06/2016). Over half of these papers have reported research carried out in North America, mostly in the United States, whereas less than 20% of this work was conducted in Europe. The majority of these papers appeared in educational journals, with a smaller proportion published in cognitive or developmental journals, and even fewer in neuroscience journals or in specialist journals on emotion or stress.

Against this backdrop, it is easier to see the contribution of this collection of papers to the literature. Most of the contributors are from European countries, and many papers deal with relatively less-investigated issues, including the relationship between MA and social influences, the measurement of MA, the physiological correlates of MA, and MA outside the classroom. The Topic also includes a number of review papers that, besides summarizing existing findings, highlight some important gaps in our current knowledge and make recommendations for future investigations. Finally, some papers present methodological innovations.

## MA research: the first 60 years and beyond

Dowker et al. summarize many of the most important MA-related findings since the first publication on the topic almost 60 years ago. Dowker et al. discuss the separability of MA from other related constructs (e.g., general- and test-anxiety, and attitudes to mathematics), as well as some issues regarding the measurement of MA. Dowker et al. also explore some other correlates of MA (e.g., physiological and socio-cultural processes), and tentative approaches to reduce or prevent MA. Finally, the paper also points to directions for future research, some of which (e.g., the relationship between gender stereotypes and MA, and the “chicken or egg” question of the link between MA and mathematics performance) were in the focus of other contributions.

## MA and basic processing of numbers

Another review paper by Artemenko et al. provides an overview of the neural correlates of MA. Artemenko et al. argue that whereas behavioral studies mostly observe an influence of MA on difficult maths tasks, neurophysiological studies show that processing efficiency is also affected in basic number processing. This conclusion is in line with Dietrich et al. who replicated previous findings by Maloney et al. (2011) and Núñez-Peña and Suárez-Pellicioni (2014) by showing a larger symbolic distance effect in maths anxious individuals. At the same time, Dietrich et al. question earlier conclusions that maths anxious individuals have a deficient approximate number system.

## MA, attentional resources, working memory, and inhibition

Artemenko et al. also discuss the neurological evidence supporting Eysenck et al.'s (2007) attentional control theory. According to this theory, attentional, and processing resources are impaired by worry, and this can only be compensated by increased cognitive effort. Four contributions within the Topic address related issues. Both Rubinsten et al. and Suárez-Pellicioni et al. present behavioral evidence for an attentional bias in MA toward threatening (i.e., maths-related) stimuli. These authors argue that this bias could lead to the exacerbation of MA, as it could result in an overestimation of the level of threat in maths-related situations. In another contribution, Núñez-Peña and Suárez-Pellicioni also present evidence from event-related potentials for attentional and processing differences between high- and low-MA individuals, while they perform multi-digit additions.

Passolunghi et al. compare the academic achievement and cognitive profiles of secondary school students with high and low MA. Passolunghi et al. show that, besides lower achievement in mathematics, students with high MA perform less well in verbal short-term memory and working memory tasks, and are less able to inhibit irrelevant information. Additionally, measures of inhibitory control and fact retrieval were the best predictors for identifying students with high or low MA. A notable methodological feature of the studies by Núñez-Peña and Suárez-Pellicioni, Passolunghi et al., and Suárez-Pellicioni et al. is that they recruited participants with particularly high/low levels of MA, and compared these extreme groups.

## The relationship between MA and maths performance

Whereas it is well-established that there is a moderate negative relationship between MA and maths performance (see Hembree, 1990 and Ma, 1999 for meta-analyses), the evidence regarding the direction of a possible causal link is mixed. In the previous sections we described evidence for the potential of MA to disrupt mathematical performance, and even very basic maths-related processing. Besides evidence for this Debilitating Anxiety Model, Carey et al. also review the findings supporting the Deficit Theory (i.e., that poor maths performance might elicit MA; cf., Tobias, 1986). Carey et al. propose that instead of trying to decide between these proposals, a Reciprocal Theory (where the causal link between MA and maths performance is bidirectional) seems most plausible. Nevertheless, they also point to the scarcity of longitudinal research that could provide further evidence for the Deficit Theory.

The only paper in the Topic dealing with Statistics Anxiety (SA; Macher et al.) also discusses the link between anxiety and performance. Macher et al. propose that although during examinations SA might disrupt performance, SA could be related to greater motivation to avoid failure, and, thus, could be positively linked to the effort invested into exam preparation. In other words, SA can both support and hinder performance, although these effects might arise at different points in time. The question of expectations about maths performance prior to testing is further addressed by Erickson and Heit.

## Identification with maths, maths confidence, and MA

Erickson and Heit investigate the link between self-confidence and actual performance in maths, biology and literature in university undergraduates. Erikson and Heit compare students' performance estimates before and after a test in each subject. Students generally overestimated their performance when they made predictions before completing the tests, but this tendency was strongest in the case of maths. This overconfidence in maths is interesting, given that Study 2 demonstrated high levels of MA in participants. Erikson and Heit point out that both MA and overconfidence could lead to avoidance behaviors, such as spending less time on exam preparation or missing classes.

Necka et al. introduce a single-item measure of self-maths overlap, modeled on Aron et al.'s (1992) the Inclusion of Other in Self Scale. Necka et al. demonstrate that self-maths overlap is negatively related to MA. Moreover, MA is more strongly related to maths performance in individuals with low self-maths overlap (i.e., in individuals who perceive maths as less self-relevant). Notably, this difference appears to be only partially explained by the tendency of individuals with high self-maths overlap to overestimate their maths ability.

Jansen et al. present a new scale to measure the tendency to use maths in everyday life, and they also investigate its relationship with maths skills, perceived maths competence and MA in a large adult sample. Jansen et al. report gender differences in all of these constructs, showing a male advantage. In both genders, perceived maths competence mediated the link between maths skills and everyday use of maths. In women only, MA was an additional mediator of the link between maths skills and everyday use of maths. This study adds to the limited literature on gender differences in the link between MA and maths performance (e.g., Devine et al., 2012; Hill et al., 2016).

## Social influences, gender stereotypes, and MA

The relationship between the development of MA and social influences, such as parents' endorsement of maths-related gender stereotypes (e.g., Tomasetto et al., 2015) is a relatively neglected issue within the MA literature. Two contributions to the Topic have investigated parental influences. Casad et al. demonstrate that parents' MA interacts with their child's MA to predict the child's self-efficacy, classroom maths performance, and maths-related attitudes.

Bosman and De Smedt further argue that MA might reflect a maladaptive affect-regulation mechanism that is characteristic of insecure attachment relationships. Their hypothesis was supported by the finding that individual differences in MA were related to insecure attachment, independent of age, sex, and IQ. Additionally, mathematics achievement was associated with insecure attachment and this effect was mediated by MA.

Bieg et al. extend previous work by Goetz et al. (2013) that showed higher trait MA in females, but no gender difference in state MA. Bieg et al. demonstrate that the discrepancy between state and trait MA (i.e., the tendency to overestimate MA) was stronger in females who endorsed the gender stereotype of maths being a male domain.

## Measurement of MA and cross-cultural validity of measurement scales

As most MA scales have been developed in North America, it is important to establish the cross-cultural validity of these instruments. Cipora et al. investigate the psychometric properties of the Polish adaptation of the Abbreviated Math Anxiety Scale (AMAS; Hopko et al., 2003), a widely used 9-item scale. Cipora et al. demonstrate high reliability, as well as very good construct, convergent and discriminant validity. This adds to previous work that demonstrated the excellent psychometric properties of the Iranian (Vahedi and Farrokhi, 2011) and Italian (Primi et al., 2014) adaptations of the AMAS.

Pletzer et al. present the psychometric analysis of a German adaptation of the MARS30-brief (Suinn and Winston, 2003), and propose that a five-factor model (including Evaluation Anxiety, Learning Mathematics Anxiety, Everyday Numerical Anxiety, Performance Anxiety, and Social Responsibility Anxiety) is the best fitting and most parsimonious representation of the factorial structure of the scale. They also establish the measurement invariance of the scale across genders, and show that gender differences are specific to Evaluation Anxiety, Learning Mathematics Anxiety, and Performance Anxiety.

## Conclusions and looking ahead to the next 60 years

Extending contributions from North American researchers, the present collection of papers brings European research into MA to the forefront, while exploring some novel and less-researched issues, such as MA and basic numerical processing; the social determinants of MA; and the links between MA, self-concept, and self-confidence.

This Topic also offers some methodological innovations, including comparisons between extreme groups of high- and low-MA participants, and new measures of self-maths overlap, as well as using maths in everyday life. Investigating MA in everyday situations could be an important direction for future research, as recent studies indicate that the effect of MA extends beyond educational contexts. Specifically, MA has been found to be linked to decision-making skills (Morsanyi et al., 2014; Silk and Parrott, 2014).

Nevertheless, there are also some topics that remained partially or wholly unaddressed by these contributions. Although the origins of MA are not well-understood, research into MA with young children remains scarce (although see e.g., Berkowitz et al., 2015; Maloney et al., 2015; Ramirez et al., 2016). Longitudinal studies with young children (e.g., Cargnelutti et al., 2016) would be particularly important for a better understanding of the origins of MA.

Further investigations into the measurement of MA are also necessary. Researchers use various instruments (ranging from single-item scales to instruments that consist of 30 or more items). The psychometric properties of these scales might differ considerably, and this can result in inconsistencies between the findings of studies. It is also common that studies with young participants use scales developed for adults, or instruments that were developed to measure MA in educational contexts are administered to adults who are no longer in education. Rolison et al. (2016) presented a scale to measure MA outside academic contexts. Investigations into MA in everyday life could make it possible to extend this work to new populations (e.g., older adults) and new contexts, such as decisions about investments, consumer behavior or lifestyle choices.

## Author contributions

All authors listed have made substantial, direct, and intellectual contribution to the work, and approved it for publication.

## Funding

The writing of this paper was supported by a Royal Society International Exchanges grant to KM and CT (reference number: IE150463).

### Conflict of interest statement

The authors declare that the research was conducted in the absence of any commercial or financial relationships that could be construed as a potential conflict of interest.

## References

[B1] Aron A. Aron E. N. Smollan D. (1992). Inclusion of other in the self scale and the structure of interpersonal closeness. J. Pers. Soc. Psychol. 63, 596–612. 10.1037/0022-3514.63.4.596

[B2] Ashcraft M. H. (2002). Math anxiety: personal, educational, and cognitive consequences. Curr. Dir. Psychol. Sci. 11, 181–185. 10.1111/1467-8721.00196

[B3] Berkowitz T. Schaeffer M. W. Maloney E. A. Peterson L. Gregor C. Levine S. C.. (2015). Math at home adds up to achievement in school. Science 350, 196–198. 10.1126/science.aac7427 26450209

[B4] Cargnelutti E. Tomasetto C. Passolunghi M. C. (2016). How is anxiety related to math performance in young students? A longitudinal study of Grade 2 to Grade 3 children. Cogn. Emot. [Epub ahead of print]. 10.1080/02699931.2016.1147421 26935005

[B5] Devine A. Fawcett K. Szûcs D. Dowker A. (2012). Gender differences in mathematics anxiety and the relation to mathematics performance while controlling for test anxiety. Behav. Brain Funct. 8:33. 10.1186/1744-9081-8-33 22769743 PMC3414752

[B6] Eysenck M. W. Derakshan N. Santos R. Calvo M. G. (2007). Anxiety and cognitive performance: attentional control theory. Emotion 7, 336–353. 10.1037/1528-3542.7.2.336 17516812

[B7] Goetz T. Bieg M. Lüdtke O. Pekrun R. Hall N. C. (2013). Do girls really experience more anxiety in mathematics? Psychol. Sci. 24, 2079–2087. 10.1177/0956797613486989 23985576

[B8] Hembree R. (1990). The nature, effects, and relief of mathematics anxiety. J. Res. Math. Educ. 21, 33–46. 10.2307/749455

[B9] Hill F. Mammarella I. C. Devine A. Caviola S. Passolunghi M. C. Szûcs D. (2016). Maths anxiety in primary and secondary school students: gender differences, developmental changes and anxiety specificity. Learn. Individ. Differ. 48, 45–53. 10.1016/j.lindif.2016.02.006

[B10] Hopko D. R. Mahadevan R. Bare R. L. Hunt M. K. (2003). The Abbreviated Math Anxiety Scale (AMAS) construction, validity, and reliability. Assessment 10, 178–182. 10.1177/1073191103010002008 12801189

[B11] Ma X. (1999). A meta-analysis of the relationship between anxiety toward mathematics and achievement in mathematics. J. Res. Math. Educ. 30, 520–540. 10.2307/749772

[B12] Maloney E. A. Ansari D. Fugelsang J. A. (2011). The effect of mathematics anxiety on the processing of numerical magnitude. Q. J. Exp. Psychol. 64, 10–16. 10.1080/17470218.2010.533278 21113858

[B13] Maloney E. A. Ramirez G. Gunderson E. A. Levine S. C. Beilock S. L. (2015). Intergenerational effects of parents' math anxiety on children's math achievement and anxiety. Psychol. Sci. 26, 1480–1488. 10.1177/0956797615592630 26253552

[B14] Morsanyi K. Busdraghi C. Primi C. (2014). Mathematical anxiety is linked to reduced cognitive reflection: a potential road from discomfort in the mathematics classroom to susceptibility to biases. Behav. Brain Funct. 10:31. 10.1186/1744-9081-10-31 25179230 PMC4166027

[B15] Núñez-Peña M. I. Suárez-Pellicioni M. (2014). Less precise representation of numerical magnitude in high math-anxious individuals: an ERP study of the size and distance effects. Biol. Psychol. 103, 176–183. 10.1016/j.biopsycho.2014.09.004 25224181

[B16] OECD (2015). The ABC of Gender Equality in Education: Aptitude, Behaviour, Confidence. Pisa: OECD Publishing 10.1787/9789264229945-en

[B17] Primi C. Busdraghi C. Tomasetto C. Morsanyi C. Chiesi F. (2014). Measuring math anxiety in Italian college and high school students: validity, reliability and gender invariance of the Abbreviated Math Anxiety Scale (AMAS). Learn. Individ. Differ. 34, 51–56. 10.1016/j.lindif.2014.05.012

[B18] Ramirez G. Chang H. Maloney E. A. Levine S. C. Beilock S. L. (2016). On the relationship between math anxiety and math achievement in early elementary school: the role of problem solving strategies. J. Exp. Child Psychol. 141, 83–100. 10.1016/j.jecp.2015.07.014 26342473

[B19] Rolison J. J. Morsanyi K. O'Connor P. (2016). Can I count on getting better? Association between math anxiety and poorer understanding of medical risk reductions. Med. Decis. Making [Epub ahead of print]. 10.1177/0272989X15602000 26296620

[B20] Silk K. J. Parrott R. L. (2014). Math anxiety and exposure to statistics in messages about genetically modified foods: effects of numeracy, math self-efficacy, and form of presentation. J. Health Commun. 19, 838–852. 10.1080/10810730.2013.837549 24479699

[B21] Suinn R. M. Winston E. H. (2003). The Mathematics Anxiety Rating Scale, a brief version: psychometric data. Psychol. Rep. 92, 167–173. 10.2466/pr0.2003.92.1.167 12674278

[B22] Tobias S. (1986). Anxiety and cognitive processing of instruction, in Self-Related Cognitions in Anxiety and Motivation, ed Schwarzer R. (Hillsdale, NJ: Lawrence Erlbaum Associates), 35–54.

[B23] Tomasetto C. Mirisola A. Galdi S. Cadinu M. (2015). Parents' math–gender stereotypes, children's self-perception of ability, and children's appraisal of parents' evaluations in 6-year-olds. Contemp. Educ. Psychol. 42, 186–198. 10.1016/j.cedpsych.2015.06.007

[B24] Vahedi S. Farrokhi F. (2011). A confirmatory factor analysis of the structure of abbreviated math anxiety scale. Iran. J. Psychiatry 6, 47–53. Available online at: http://ijps.tums.ac.ir/index.php/ijps/article/view/283 22952521 PMC3395944

